# Development and Efficacy of the Antivenom Specific to Severe Envenomations in Morocco and North Africa: Advancements in Scorpion Envenomation Management

**DOI:** 10.3390/toxins16050214

**Published:** 2024-05-04

**Authors:** Bouchra Darkaoui, Ines Hilal, Soukaina Khourcha, Ayoub Lafnoune, Salma Chakir, Ayoub Aarab, Abdellah Moustaghfir, Ouafaa Aniq Filali, Naoual Oukkache

**Affiliations:** 1 Laboratory of Venoms and Toxins, Pasteur Institute of Morocco, 1 Place Louis Pasteur, Casablanca 20250, Morocco; darkaoui.bouchra@hotmail.com (B.D.); drineshilal@gmail.com (I.H.); khourcha.soukaina9@gmail.com (S.K.); ayoublafnoune@gmail.com (A.L.); salmachakir19@gmail.com (S.C.); 2 Laboratory of Molecular Genetics, Physiopathology and Biotechnology, Faculty of Sciences Ain Chock, Hassan II University of Casablanca, B.P 5366 Maarif, Casablanca 20000, Morocco; ouafa.aniqfilali@gmail.com; 3 Laboratory of Anatomical Pathology Marrakech, Agadir 80000, Morocco; ayoub-aarab@hotmail.fr; 4 Laboratory of Research Odontological, Biomaterials and Nanotechnology, Department of Fundamental Sciences, Faculty of Dental Medicine, Mohammed V University in Rabat, B.P 6212 Madinat Al Irfane, Rabat 10000, Morocco; moustaghfir@um5r.ac.ma

**Keywords:** scorpion venom, toxicity, antivenom, immunization, neutralization, cross-reactivity

## Abstract

Scorpion envenomation poses a global public health issue, with an estimated 1,500,000 cases worldwide annually resulting in 2600 deaths. North Africa, particularly Morocco, experiences severe envenomations, mainly attributed to *Androctonus mauretanicus* and *Buthus occitanus* in Morocco, and *Buthus occitanus* and *Androctonus australis hector* in Algeria and Tunisia, with case numbers often underestimated. Current treatment relies mainly on symptomatic approaches, except in Morocco, where management is limited to symptomatic treatment due to controversies regarding specific treatment. In Morocco, between 30,000 and 50,000 scorpion envenomation cases are reported annually, leading to hundreds of deaths, mainly among children. Controversies among clinicians persist regarding the appropriate course of action, often limiting treatments to symptomatic measures. The absence of a specific antivenom for the venoms of the most lethal scorpions further exacerbates the situation. This study aims to address this gap by developing a monovalent antivenom against the endemic and most dangerous scorpion, *Androctonus mauretanicus*. The antivenom was produced by immunizing albino rabbits with a mixture of *Androctonus mauretanicus* venom collected from high-risk areas in Morocco. Immunizations were performed by subcutaneous injections at multiple sites near the lymphatic system, following an immunization schedule. Production control of neutralizing antibody titers was conducted through immunodiffusion. Once a sufficient antibody titer was achieved, blood collection was performed, and the recovered plasma underwent affinity chromatography. The efficacy of purified IgG was evaluated by determining the ED_50_ in mice, complemented by histological and immunohistochemical studies on its ability to neutralize venom-induced tissue alterations and the neutralization of toxins bound to receptors in the studied organs. The monovalent antivenom demonstrated specificity against *Androctonus mauretanicus* venom and effective cross-protection against the venom of the scorpions *Buthus occitanus* and *Androctonus australis hector*, highly implicated in lethal envenomations in the Maghreb. This study shows that the developed monovalent antivenom exhibits notable efficacy against local scorpions and a surprising ability to neutralize the most lethal envenomations in North Africa. These results pave the way for a new, more specific, and promising therapeutic approach to countering severe scorpion envenomations, especially in Morocco, where specific treatment is lacking.

## 1. Introduction

Scorpion envenomations constitute a public health problem in tropical and subtropical regions, particularly in North Africa. Globally, zoologists have documented nearly 1600 species of scorpions [1,2]. Each year, there are approximately 1,500,000 cases of scorpion stings worldwide, resulting in 2600 deaths, mainly attributable to the Buthidae species, which are regarded as the most extremely dangerous [3].

In Morocco, this family represents 88% of the scorpionic fauna, with *Androctonus mauretanicus* (*Am*) and *Buthus occitanus* (*Bo*) standing out as the species most involved in severe scorpion envenomations [4,5,6]. Even on the scale of the North Africa, the *Bo* is known for its wide distribution also in Algeria, Tunisia and Egypt. In addition, the specie of *Androctonus australis hector* (*Aah*) is considered one of the most armful scorpions in the world, with a distribution mainly in North Africa (Algeria, Libya, Tunisia and recently found in Morocco) [7,8]. Epidemiological data record between 25,000 and 40,000 scorpion stings annually in Morocco, most of which significantly affect children under 15 years of age [9,10]. In Algeria, an average of 50,000 cases of scorpion stings are recorded annually, of which 2.5% are severe envenomations, resulting in 100 to 150 deaths. Children between 5 and 14 years of age are most affected in terms of mortality [11]. In Tunisia, 30,000 epidemiological cases are reported annually, of which 1000 include systemic manifestations requiring hospitalization with a high mortality rate in children (15.1%) compared to adults (5.6%) [12,13].

Scorpion venom is a water-soluble, heterogeneous, and antigenic mixture. These venoms are used by scorpions to attack and capture their prey [14]. The composition of the venom includes mucopolysaccharides, hyaluronidase, phospholipase, serotonin, histamine, enzyme inhibitors, and proteins in the form of neurotoxic peptides [15]. These neurotoxic peptides are distinguished by their diversity, both in terms of their molecular polymorphisms and the diversity of their target receptors at the level of the ion channels of neuronal membranes. Thus, depending on their target, four large families of toxins are currently identified: those acting on sodium, potassium, chlorine, and calcium channels [16]. Although the inherent toxicity of scorpion venom induces a plurality of more marked clinical and pathophysiological effects, this complexity highlights the multifactorial nature of bodily responses to envenomation. The variety of clinical manifestations and physiological disturbances reflects the diversity of bioactive components present in the venom; each element interacts in a specific manner with the body’s biological systems, thereby triggering a wide range of pathological reactions [15,17,18,19]. The use of scorpion antivenom is surrounded by some controversy, according to the World Health Organization (WHO) guidelines [20]. These controversies pose notable challenges in terms of the development of antivenom and therapeutic management [21]. They arise generally from several parameters, which are either linked to the high variability and complexity of the biochemical composition of the venom or linked to the antivenom and its composition, concentration, pharmacokinetics, and conditions of administration. The aspects cited influence its effectiveness, while the variability in individual patient responses further complicates the situation. A thorough understanding of all these elements is crucial for optimizing the use of antivenom in the treatment of scorpion envenomations [22]. Although immunotherapy has demonstrated its effectiveness in various countries, it remains a particularly hotly debated issue in Morocco [22,23,24]. Indeed, the development of an effective antivenom requires local production to adapt to the species of scorpions present in Morocco, and this requirement underlines the need to adapt therapeutic solutions to local scorpion fauna.

In Morocco, the non-use of specific treatments is a complex issue that has sparked debates and controversies. Several factors contribute to this situation, including concerns about the efficacy, safety, and accessibility of commercialized antivenoms. The intraspecific and interspecific variability of scorpion venoms complicates the development of tailored antivenoms. This venom diversity can affect the neutralization of specific toxins and influence patients’ immune response, making it difficult to create universal or widely effective antivenoms. This complexity underscores the need for targeted translational approaches in antivenom development. In this context, this work aims to develop an antivenom against *Androctonus mauretanicus*, a species involved in the deadliest envenomations in Morocco, and to evaluate its efficacy in vivo by determining the effective dose 50 (ED_50_), neutralizing tissue alterations, and neutralizing toxins bound to the receptors of the studied organs. In addition, we examined the antigenic reactivity of the antivenom produced against *Buthus occitanus* (*Bo*) and *Androctonus australis hector* (*Aah*), species of Buthidae scorpion responsible for fatal envenomations in the North Africa.

## 2. Results

### 2.1. Quality Control of the Scorpion Venom

#### 2.1.1. Protein concentration

The results showed that the *Am* venom was rich in protein with a concentration equal to 188.5 ± 3.39 mg/mL.

#### 2.1.2. SDS-PAGE Profile

**Figure 1:**
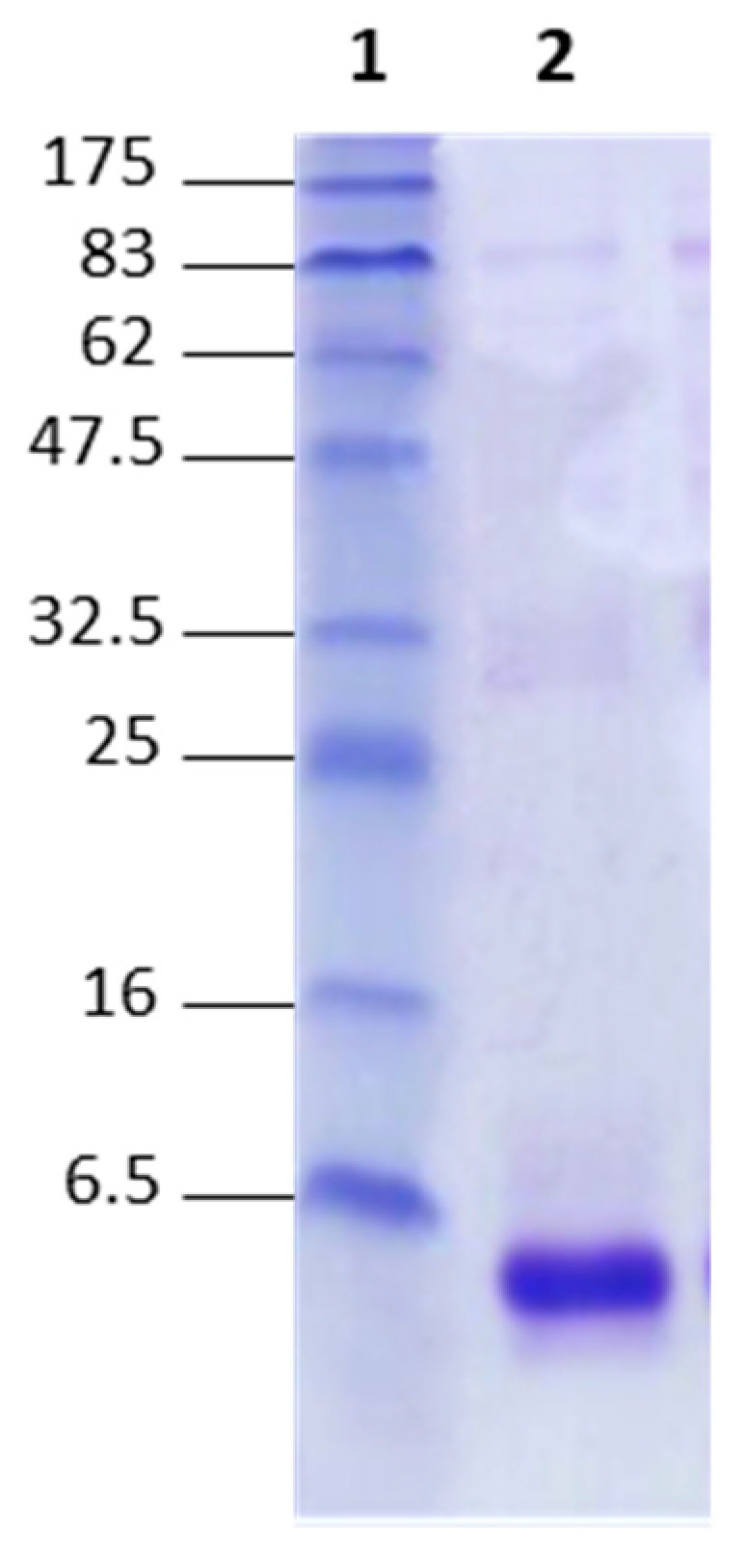
SDS-PAGE profile of *Am* scorpion venom. Lane 1 contains molecular weight markers in kDa; Lane 2 contains *Am* venom.

The analysis of the electrophoretic profile showed the presence of bands with high intensity and a molecular weight lower than 6.5 kDa, corresponding to the molecular weights of scorpion toxins (Figure 1). This result indicates that the composition of scorpion venom is rich in molecules of less than 6.5 kDa, which are mainly toxins.

#### 2.1.3. The Median Lethal Dose (LD_50_) and Sublethal Doses (sLD) of Scorpion Venom

The recorded median lethal dose (LD_50_) and sublethal dose (sLD) of *Am* scorpion venom were 337 ± 45.7 and 177 ± 3.8 µg/kg body weight, respectively, following intraperitoneal injection.

### 2.2. Antivenom Production

#### 2.2.1. Control of Antivenom Production

**Figure 2:**
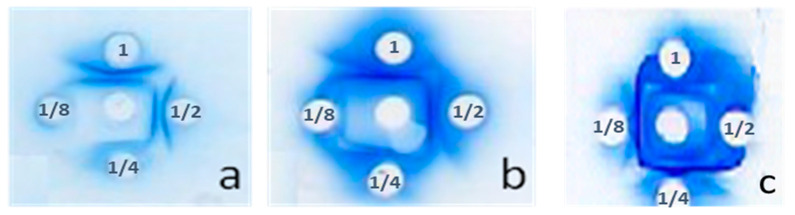
Double immunodiffusion illustrating the precipitation arcs resulting from the interaction between the *Am* venom (in the central well) and the neutralizing antibodies of the rabbit plasma (in the peripheral wells), indicating the production of antibodies. The peripheral wells contain increasing dilutions of rabbit plasma collected on days 21 (**a**), 35 (**b**), and 42 (**c**) after the first immunization.

The results of the immunodiffusion test showed the presence of precipitation arcs (antigen–antibody reactions), indicating the production of specific neutralizing antibodies against *Am* scorpion venom in the immunized rabbits. Depending on the post-immunization time, the precipitation lines became more pronounced (Figure 2a–c), indicating strong production of neutralizing antibodies.

**Figure 3:**
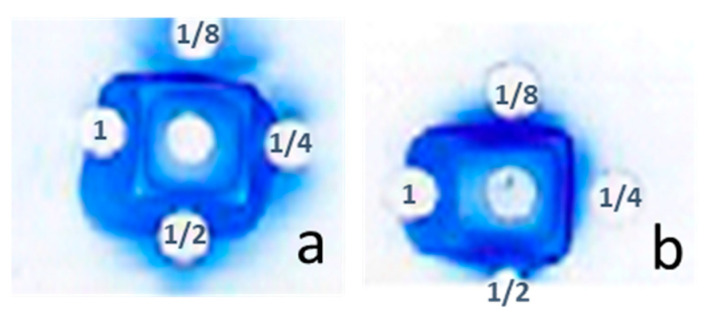
Double immunodiffusion illustrating the precipitation arcs resulting from the interaction between the *Bo* (**a**) and *Aah* (**b**) venoms (in the central well) and the neutralizing antibodies of the rabbit plasma (in the peripheral wells), indicating the production of antibodies. The peripheral wells contain increasing dilutions of rabbit plasma collected on day 42 after the first immunization.

Figure 3 showed cross-reaction between the produced antibodies and the venom of scorpions *Bo* and *Aah* (Figure 3a,b), indicating the presence of the same common antigenic determinants. The immune response was higher for *Bo* venom than for *Aah* venom.

#### 2.2.2. Purification of Specific Antibodies

**Figure 4:**
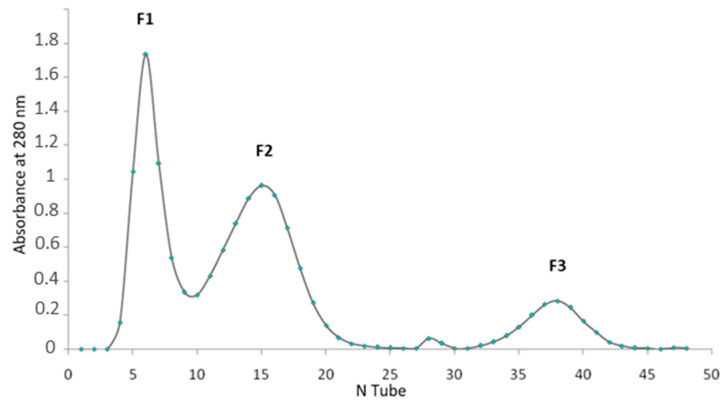
Chromatogram of the purification profile of specific antibodies obtained by affinity chromatography.

The neutralizing antibodies developed against *Am* scorpion venom were purified from the serum of immunized rabbits using affinity chromatography. Three fractions were obtained (Figure 4): fraction 1 contained the non-retained fraction containing non-specific plasma proteins, and fraction 2 contained the specific antibodies with an affinity for *Am* venom which was different from that in fraction 3. The concentration of the specific immunoglobulin from fraction 2, which underwent efficacy evaluation, was approximately 0.11 mg/mL with a pool volume of 12 mL, resulting in a protein amount equivalent to 1.32 mg. 

#### 2.2.3. Control of Antibody Purity

**Figure 5:**
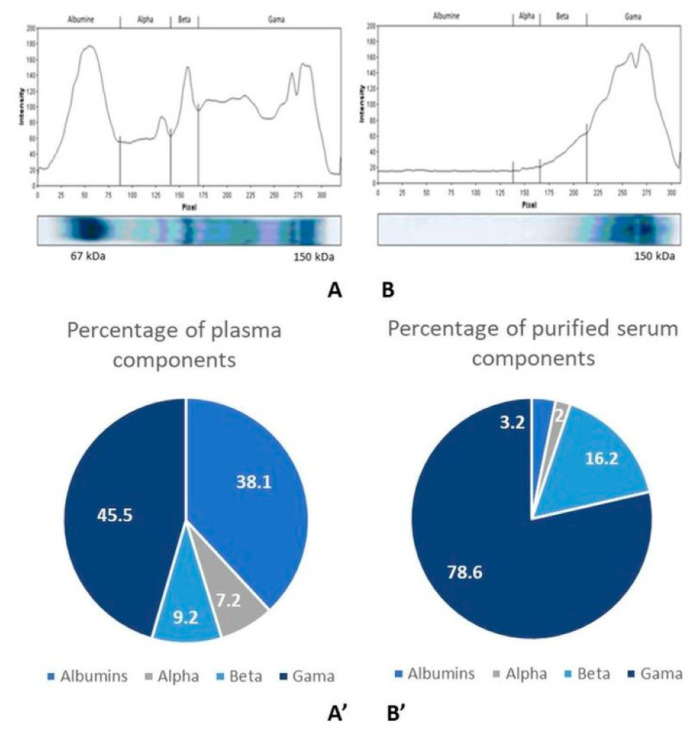
SDS-PAGE electrophoresis and component percentage of the plasma (**A**,**A’**) and purified serum (**B**,**B’**).

Figure 5 depicts the electrophoretic analysis of plasma and antibodies from fraction 2 purified by affinity chromatography. It shows that these are indeed IgG gammaglobulins with a molecular weight of 150 kDa, indicating that our antibodies are pure and well purified, with a dense band corresponding to gammaglobulins. In contrast, the plasma shows a dense band corresponding to albumins (Figure 5).

#### 2.2.4. Calculation of Neutralizing Antibody Yield

The concentrations of plasma and purified serum were equal to 14.06% and 8.65%, respectively, with the yield equal to 8.2% (Table 1).

#### 2.2.5. The Cross-Reactivity of the Produced Antivenom

**Figure 6:**
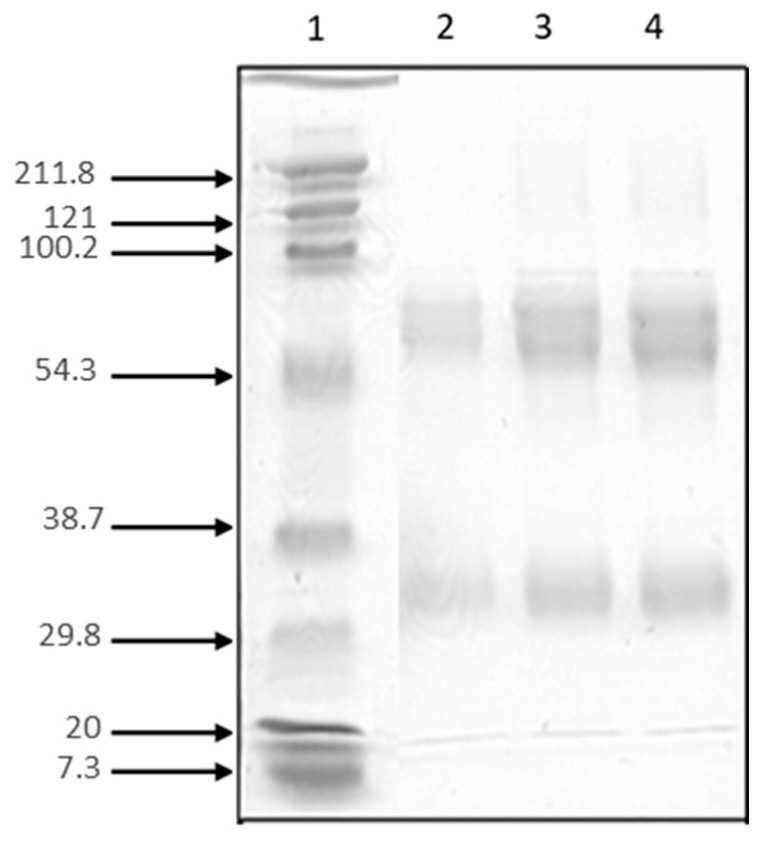
Western blotting using the produced antivenom against the *Am*, *Bo* and *Aah* venom. Lane 1 contains molecular weight markers in kDa; Line 2 contains *Am* venom; Lane 3 contains *Bo* venom; Lane 4 contains *Aah* venom.

Western blotting profiles showed that the generated antibody presented a cross-reaction of multiple components present in the crude venom of the two other species of scorpion, *Bo* and *Aah* (Figure 6). These results suggested the presence of common peptides in the *Bo* venom compared to the *Aah*.

#### 2.2.6. Neutralizing Effect of Antivenom Developed against *Am* Venom on *Bo* and *Aah* Scorpion Venoms: A Study of Effective Doses (ED_50_)

The determination of the effective doses (ED_50_) of the antivenom revealed its efficacy in neutralizing the lethal effect induced by the 3LD_50_ of each injected venom (Table 2). The antivenom developed against the *Am* venom demonstrated high effectiveness, with a dose of 47.3 µL, which is understandable given that this venom led to its production. This was then followed by the *Bo* venom (51.86 µL) and the *Aah* venom (64.9 µL). These results consistently demonstrated that the antivenom produced exhibits cross-reactivity with the *Bo* and *Aah* scorpion venoms, even though it is not produced against these venoms.

### 2.3. Neutralization of Tissue Alterations in the Studied Organs (Brain and Heart)

#### 2.3.1. Histological Analysis of Brain Tissue Alterations Caused by the Venoms and Neutralized by the Developed Antivenom

**Figure 7:**
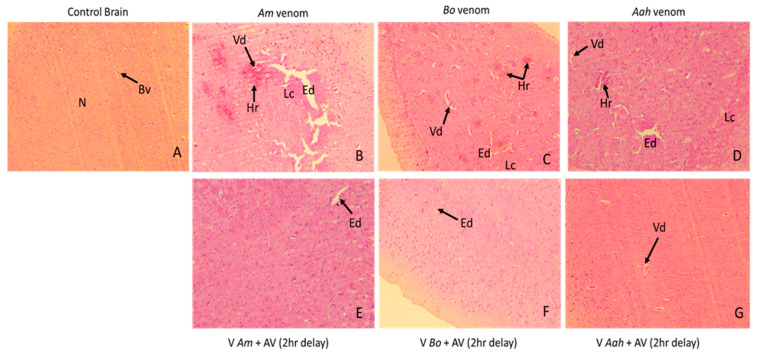
Antivenom neutralization of tissue alterations induced in brain tissue by *Am*, *Bo* and *Aah* scorpion venoms (×10). (**A**): Image corresponds to the control group. (**B**–**D**): Images correspond to the groups treated with the scorpion venoms. (**E**–**G**): Images correspond to groups treated with the antivenom 2 h delay. Bv: blood vessel; N: neuron; Vd: vasodilatation; Ed: edema; Lc: loss of cellularity; Hr: hemorrhage.

The various alterations generated by the scorpion venoms were observed in the histological sections and were characterized by varying degrees of vasodilation, hemorrhage, and edema in all treated mouse groups, with a loss of cellularity mostly in mice injected with *Am* and *Aah* venoms compared to *Bo* venom (Figure 7B–D). *Am* venom exhibited the highest intensity of all observed brain alterations. Administration of the produced antivenom with a two-hour delay largely neutralized the previously observed damage. However, discreet edema persisted following the effects of *Am* and *Bo* venoms, and vasodilation was observed in the group of mice treated with *Aah* venom (Figure 7E–G).

#### 2.3.2. Histological Analysis of the Cardiac Tissue Alterations Caused by the Venoms and Neutralized by the Developed Antivenom

**Figure 8:**
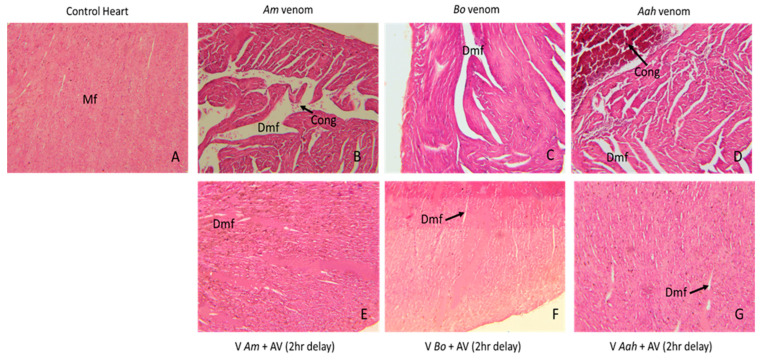
Antivenom neutralization of tissue alterations induced in cardiac tissue by *Am*, *Bo* and *Aah* scorpion venoms (×10). (**A**): Image corresponds to the control group. (**B**–**D**): Images correspond to the groups treated with the scorpion venoms. (**E**–**G**): Images correspond to groups treated with the antivenom 2 h delay Mf: muscle fiber; Dmf: degeneration of myocardium; Cong: congestion.

The cardiac tissue was significantly affected by the venom of each of the different scorpions, *Am*, *Bo*, and *Aah*. Histological sections revealed more extensive myocardial degeneration in the group injected with *Am* venom compared to the other two. A more pronounced congestion was observed in the *Aah* venom group than in the *Am* venom group (Figure 8B–D). Our results indicated that after injection of the developed antivenom, the tissue structure of the heart improved significantly, with a reduction in myofiber-related degeneration in all three groups of mice envenomed by *Am*, *Bo* and *Aah* (Figure 8E–G).

### 2.4. Immunohistological Profile: Neutralization of Toxins Bound to Receptors in the Studied Organs (Brain and Heart)

#### 2.4.1. Neutralization of Toxins Bound to Receptors in Brain Tissue

**Figure 9:**
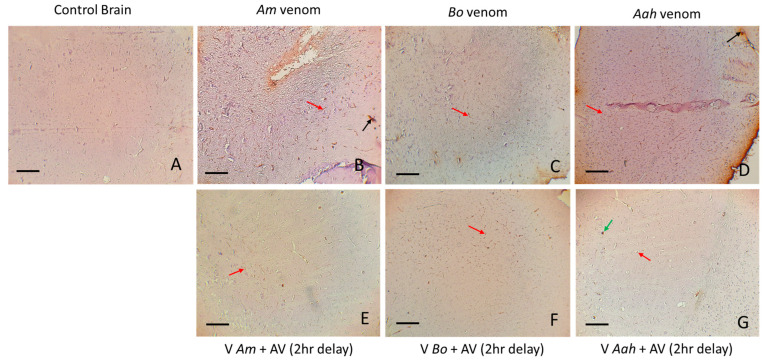
Antivenom neutralization of toxins fixed to tissue receptors at the level of heart tissue by the effect of the venoms of *Am*, *Bo* and *Aah*. (**A**): The control group. (**B**–**D**): Groups treated with the scorpion venoms. (**E**–**G**): Groups treated with the antivenom 2 h delay. Calibration bar = 50 µm. Brown areas are positive for venom and complex venom–antivenom. Detection in nerve cells (red arrow), vascular endothelial cells (black arrow), and inflammatory cells (green arrow).

Immunohistological analysis of the brain tissue of envenomed mice showed that scorpion venom toxins were immunohistologically detected at the level of brain cell receptors in all groups of mice envenomed with *Am*, *Bo*, and *Aah* venoms in addition to staining in endothelial and inflammatory cells in mice envenomed with *Am* and *Aah* venoms. Cytoplasmic and membrane staining were noted in all groups except those treated with *Aah* venom. Furthermore, venom toxins from *Am* venom were detected with a high percentage (70%) in brain tissue, followed by *Aah* and *Bo* venoms. After administration of the antivenom, we observed a reduction of 30%, 20%, and 40% in staining percentage in groups of mice treated with *Am*, *Bo*, and *Aah* venoms, respectively (Table 3, Figure 9).

#### 2.4.2. Neutralization of Toxins Bound to Receptors in Cardiac Tissue

**Figure 10:**
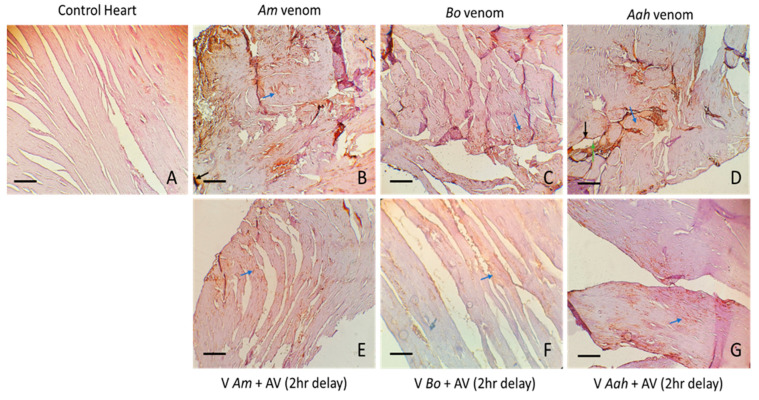
Antivenom neutralization of toxins fixed to tissue receptors at the level of heart tissue after the effect of the venoms of *Am*, *Bo* and *Aah*. (**A**): The control group. (**B**–**D**): Groups treated with the scorpion venoms. (**E**–**G**): Groups treated with the antivenom 2 h delay. Calibration bar = 50 µm. Brown areas are positive for venom and complex venom–antivenom. Detection in cardiac cells (blue arrow), vascular endothelial cells (black arrow), and inflammatory cells (green arrow).

The immunohistochemical profile revealed the presence of venom molecules in myocardial cells of mice envenomed with *Am*, *Bo* and *Aah* venoms as well as in endothelial cells in groups treated with *Am* and *Aah* venoms. Inflammatory cells were stained only in the group envenomed with *Aah* venom. Cytoplasmic and membrane staining were observed in all groups. In terms of percentage, the *Am* venom showed the highest staining intensity at 90%, followed by *Aah* and *Bo* venoms at 70% and 60%, respectively. The introduction of the antivenom significantly reduced the staining, indicating the neutralization of venom toxins bound to cardiac tissue by the neutralizing antibodies present in the developed antivenom (Table 3, Figure 10).

This neutralization also highlights the cross-reactivity of the antivenom developed against *Am* venom, which is capable of neutralizing tissue alterations and toxins bound to receptors in the studied organs.

## 3. Discussion

Envenomation through scorpion stings poses a considerable threat to public health on a worldwide scale, especially in tropical and subtropical regions. Different species of scorpions are found in North Africa, including *Androctonus mauretanicus*, *Buthus occitanus* and *Androctonus australis hector*, all of which pose potential dangers due to the severity of scorpion stings and debates surrounding immunotherapy, especially in Morocco. The present study focused on the processes involved in producing the monovalent antivenom against the endemic scorpion *Am* and testing its neutralizing effectiveness, in addition to the possibility of cross-reactivity with *Bo* and *Aah* scorpion venoms.

Quality control of the *Am* venom revealed its protein richness, which is consistent with its high toxicity. This richness in low-molecular-weight proteins of less than 14 kDa corresponds to the toxins in the venom. The electrophoretic profile confirmed this protein’s composition and showed that more than 70% of the composition of scorpion venom is toxic, while the toxins fell into two categories: long toxins (which act on the peripheral and central nervous system, causing toxicity followed by death in mice) and short toxins (which act mainly on the central nervous system, causing toxicity without death in mice). The results of LD_50_ determined by the IP route showed that the molecules responsible for mortality have a very low molecular weight (<10 kDa), enabling them to circulate rapidly in the blood. Our results are consistent with previous studies, such as that of Oukkache et al., which confirmed the high toxicity of the scorpion venom *Am* [25].

Regarding the composition of neurotoxic scorpion venom in proteins of low molecular wight and poor antigenic properties [26,27], the use of adjuvants is recommended in the production of antivenoms. Freund’s Complete Adjuvant (FCA) and Freund’s Incomplete Adjuvant (FIA), which were adopted in the present study, are widely known for their efficacy in amplifying the immune response [17]. The choice to use these adjuvants is in agreement with the immunization procedure reported by Ozkan O. et al.’s study [28]. The crude venom was used as an antigen on the basis that venom toxicity broadly results from the synergic effect of various constituents and not from a highly toxic individual component [29]. Using the same classical approach, another study produced a scorpionic antivenom [28]. After animal immunization procedures, the presence of antibodies produced against *Am* venom was detected following the respective stimuli injected with a time-dependent production rhythm; the results were confirmed by analyzing the immunodiffusion patterns three times during the immunization program. After the purification processes, the IgG antibodies, as the final product generated, revealed a cross-neutralizing reaction against the *Bo* and *Aah* venom, in which the venom of the scorpions studied shared a degree of similarity, leading to epitope recognition. The theoretical mechanisms related to cross-reactivity were discussed in the study of Ledsgaard and al., in which the ability to bind multiple different antigens can be explained by the immune tolerance of the antibodies, considering a degree of variability in antigens’ and toxins’ epitopes [30]. In terms of potency, the produced antivenom was able to neutralize 63.4 LD_50_ of the *Am* venom, and by cross-reaction, it neutralized 57.8 and 46.2 LD_50_ of the *Bo* and *Aah* venoms, respectively. The histological lesions caused by sublethal doses of each scorpion venom and neutralized by the administration of the produced antivenom were evaluated at the brain and heart level in mice. Intense tissular damage was observed following the effect of the *Am* venom and was highly neutralized by the antivenom, with slight alterations represented by discreet edema and vasodilatation in the brain and degeneration of the myocardium in the heart. The tissular structure was also generally improved in the groups treated with the produced antivenom 2 h after inoculation with *Bo* or *Aah* venom. We thus observed that the venom could be localized in nerve/myocardial, endothelial, and inflammatory cells, with significantly higher percentages in the sections of nervous and cardiac tissues treated only with the venom compared to the groups of mice treated with the antivenom administered 2 h earlier. The neutralizing effects of the antivenom reveled in these results align with numerous studies [31,32,33,34,35]. In a previous study carried out by our team, we demonstrated the effectiveness of a selective polyvalent antivenom in completely or partially neutralizing the histological alterations induced by *Am*, *Aah,* and *Bo* scorpion venoms in the main organs of mice [36]. However, for comparison purposes, the produced serum exhibited a high capacity for neutralization, especially for the venom produced against it (*Am*) compared to the selective polyvalent antivenom manufactured against various species of scorpions, including the *Am* venom [36].

The antivenom’s ability to cross-reactivate with other scorpion venoms can help in light of inter and intraspecific venom variability and the limits of the number of venoms that can be used in the immunization process.

The process of producing and purifying an antivenom against the most dangerous Am species in Morocco has been developed, and by studying the antivenom’s neutralization capabilities in vivo, we were able to respond to controversies related to the achievement of an effective antivenom at the tissue level and the neutralization of induced tissue alterations, even after 4 h of scorpion envenomation, in mice. In the same context, Boyer’s study showed the ability of the antivenom to remove venom from the plasma compartment in less than an hour by comparing with a placebo group over more than 4 h. The time taken to cure was minimal (less than 4 h) in all patients receiving antivenom (compared to 15% in the control group) [37]

These promising results underline the effectiveness of immunotherapy against the scorpion envenomations caused mostly by dangerous scorpion species in North Africa, namely *Androctonus mauretanicus*, *Buthus occitanus* and *Androctonus australis hector*. Due to the neutralizing and curative properties demonstrated, immunotherapy can thus be considered a specific treatment to be recommended in Morocco in complementarity with a symptomatic approach. Furthermore, various studies have indicated that patients receiving treatment combining prazosin and a specific antivenom, whether adults or children, adapted to the clinical severity of the envenomation, showing a faster recovery compared to those treated only with prazosin. In addition, this combined treatment is well tolerated [38].

An antivenom with approved neutralizing performance in preclinical and clinical phases is intended for production and utilization in the treatment of scorpion envenomations. Despite the efficacity of the immunotherapy, there are practical challenges involved in its distribution to ensure rapid administration of antivenom and improve the clinical outcomes of patients.

## 4. Conclusions

In this study, a monospecific antivenom was successfully produced against the endemic and most dangerous species in Morocco, *Androctonus mauretanicus*. Its efficacity and in vivo neutralization were approved regarding tissue damage and in vivo localization at the brain and heart level in the presence or absence of the antivenom. Additionally, a cross-reactivity was noted with *Bo* and *Aah* scorpion venoms, as applied in severe cases of envenomation in North Africa. Since immunotherapy has demonstrated its effectiveness as a therapeutic approach, there is an urgent need to adopt large-scale production, especially in Morocco.

## 5. Materials and Methods

### 5.1. Scorpion Venoms

High-risk *Androctonus mauretanicus* (*Am*) and the *Buthus occitanus* (*Bo*) scorpions were collected from Morocco and kept at the scorpionarium of the Pasteur Institute of Morocco. The venoms were extracted by the electrical stimulation method [39] and centrifuged at 14,000 r/min at 4 °C and for 20 min. The lyophilized form of the *Androctonus australis hector* (*Aah*) Tunisian scorpion venom was generously provided by ATheris Laboratories. Their median lethal dose (LD_50_) and sublethal dose (sLD) were defined, respectively, as 890.1 and 415 µg/kg for the *Bo* venom and 597 and 255 µg/kg for the *Aah* venom [36].

### 5.2. Quality Control of Scorpion Venom

#### 5.2.1. Protein Concentration

The venom protein concentration was determined by absorbance measurement at 280 nm, applying a Quartz cuvette with an optical path of 1 cm; one unit of absorbance corresponds to a protein concentration of 1 mg/mL [40,41].

#### 5.2.2. SDS-PAGE Analysis

The electrophoretic profile of the venom was obtained using 15% polyacrylamide gel with SDS under denaturing conditions (2-mercaptoethanol) according to the Laemmli method [42]. Then, 10 µL of *Am* venom was diluted in a simple buffer (10% glycerol, 2.5% SDS, 50 mM Tris-HCl, pH6.8, 5% 2-mercaptoethanol and 0.02% bromophenol blue). The mixture was denatured for 5 min in 100 °C, centrifuged at 18,000g for 25 s, and then deposited on polyacrylamide gel in addition to the protein markers (6.5–175 kDa) in a Bio-Rad system. The migration was stopped after two hours, and the gel was stained using Coomassie Blue G250 and subsequently washed with a solution of 40% methanol and 10% acetic acid.

#### 5.2.3. Determination of Median Lethal Dose (LD_50_) and Sublethal Doses (sLD) of Venom

The median lethal dose (LD_50_) of venom was determined as recommended by the World Health Organization [43]. Groups of 6 male Swiss mice (18–22 g) were used per dose of venom injected intraperitoneally. Percent mortality was recorded 24 h later. The LD_50_ was identified using the software package GraphPad Prism 7 according to the algorithm provided. The non-linear curve was traced by respecting the four-parameter logistical equation and by establishing constraints on the bottom (0% mortality) and top (100% mortality) values. The sublethal dose (sLD) was obtained by injecting decreasing amounts of *Am* venom into groups of mice [44]. In all experiments, the injections were made by the intraperitoneal route, with a total volume injected equal to 500 µL.

### 5.3. Antivenom Production

#### 5.3.1. Venom Preparation

Crude *Am* venom filtered through 0.22 µm filters was used as an antigen for the antivenom production.

#### 5.3.2. Rabbit Immunization

Table 4 presents the rabbit immunization schedule. For the first injection, Freund’s Complete Adjuvant (FCA) was used to stimulate the cellular immune system. The second, third, and fourth injections were performed with Freund’s Incomplete Adjuvant (FIA) to stimulate the humoral immune system. Subsequent injections were performed with physiological saline solution (NaCl 0.9%). A group of 4 albino rabbits (3–3.5 kg) were used, while the 5th served as a control. Injections of an increasing dose of venom were administered subcutaneously (SC) into the animal’s neck, close to the lymph nodes, according to the established immunization schedule. After each injection, lymph node formation was monitored as a sign of immune system stimulation.

#### 5.3.3. Production Control of Antibody by Immunodiffusion Test

Blood samples were taken at 21, 35, and 42 days after the first immunization to control the antibody production and decide when to bleed for collection. The technique used to assess neutralizing IgG production was the immunodiffusion test following the Ouchterlony’s method [45]. Then, 1% agarose gels were prepared on glass slides and diluted in phosphate-buffered saline (PBS). Five wells were made (one central and four around the center). Then, 15 µL of crude *Am* venom (5 mg/mL) was added to the central well, and serum diluted in series (1, 1/2, 1/4 and 1/8), obtained by centrifugation of the blood, was added to the four peripheral wells. The slides were incubated at room temperature for 24 to 48 h. If neutralizing antibodies were present, white precipitation arcs appeared, stained with Coomassie Blue R250. Our results showed that on day 42 after immunization, the serum had a good titer of neutralizing antibodies produced. The same procedure was used for *Bo* and *Aah* venoms to determine whether the antibodies produced were capable of neutralizing the toxins of these venoms by cross-reactivity or para-specificity.

#### 5.3.4. Blood Collects

Based on our results, we decided to proceed with the blood collection on the day 45 of immunization. The serum was obtained by a centrifugation of the blood samples at 3000 r/min for 10 min. The purity of the purified antibodies was checked, and the yield of purified antibodies was measured.

#### 5.3.5. Purification of Specific Antibodies by Affinity Chromatography

The first purification of the antibodies was made by ammonium sulfate precipitation at 29%, before the affinity chromatography step in which the *Am* scorpion venom was dissolved in 0.1M sodium bicarbonate; the solid-phase affinity columns contained the venom coupled to cyanogen bromide-activated Sepharose 4B. After centrifugation at 3000 r/min for 20 min, the sergeant recovered was incubated overnight at room temperature. The column was loaded with the serum collected. Then, the non-specific proteins were removed through multiple wash cycles with Tris HCl 100 mM + NaCl 0.5 M, pH 8. By adding the elution buffer (acetic acid 0.1M, pH 2.1), the targeted antibodies were eluted and neutralized immediately in tubes containing Tris HCl 1M, pH 8 [46].

#### 5.3.6. Control of Antibody Purity by Electrophoresis

Unpurified plasma and antibodies obtained from serum purification by affinity chromatography were subjected to electrophoresis using 12% polyacrylamide gel with SDS under non-reducing conditions (2-mercaptoethanol not used) while following the Laemmli method [42]. The relative abundance was obtained using the Gel Analyzer program.

#### 5.3.7. Protein Concentration of Neutralizing Antibodies

The measurement of the protein content of neutralizing antibodies was carried out according to the Bradford quantification method [47], by using bovine serum albumin (BSA) solution to obtain the calibration curve (from 0 to 10 µg/µL). The absorbance was measured at 595 nm.

#### 5.3.8. Western Blot Analysis

The *Am*, *Bo* and *Aah* scorpion venoms were separated by electrophoresis (SDS-PAGE). The gel containing the migrated venom proteins was transferred to a nitrocellulose membrane and incubated with TBS (tris-buffered saline) at 4 °C overnight. The membrane was washed three times with TBS and then incubated with the antivenom at room temperature for 1 h. After three further washes with TBS, the nitrocellulose membrane was incubated with a peroxidase conjugated antibody for 1 h at room temperature, then washed again with TBS for 10 min. Finally, the substrate diamino benzidine (DAB) was added to visualized the protein bands, which were stained with Coomassie Blue R250 [48].

#### 5.3.9. Determination of the Effective Doses (ED_50_) of the Antivenom

A fixed amount (3LD_50_) of each venom was incubated with varying volumes of antivenoms for 30 min at 37 °C. Each mixture (0.5 mL) was injected intraperitoneally to six male Swiss mice (18–22 g), and deaths were recorded up to 24 h. Controls received 3DL_50_ of venom without antiserum. Results were analyzed by the GraphPad Prism 7 Software package with a variable-slope dose–response curve and non-linear analysis. The ED_50_ obtained is defined as the volume of antivenom (in µL) able to protect half a population of mice against a specific dose of scorpion venom (3LD_50_) or as the number of LD_50_ of venom that was neutralized by 1 mL of antivenom.

#### 5.3.10. Histological Analysis of Brain Tissue Alterations Caused by the Venoms and Neutralized by the Developed Antivenom

For each venom, two groups of four male Swiss mice (18–22 g) were used. The first group were received a sublethal dose (sLD) of each crude venom. In the second one, the mice were injected with the effective dose (ED_50_) of the antivenom 2 h before the injection of the sLD of scorpion venom. All injections were made by the intraperitoneal route, with 500 µL as a total volume injected per mouse. The control group received a physiological saline solution (NaCl 0.9%). The animals were sacrificed 4 h later.

The carefully extracted brain and heart organs were immersed immediately in formaldehyde solution at 10% for 24 h; then, the tissues were dehydrated using an ethanol series and clarified with xylene. Next, they were embedded in paraffin and sliced at 4 μm thickness to be stained with hematoxylin and eosin for photonic microscope visualization [49].

#### 5.3.11. Immunohistological Profile: Neutralization of Toxins Bound to Receptors in the Studied Organs (Brain and Heart)

The brain and heart tissue sections were removed from paraffin and cycles of rehydration were executed for 5 min each using the xylol and alcohol series. The protocol of the immunohistological study was previously described for the both organs obtained from the control group, who were treated only with the scorpion venom, and the other group, who were treated with the antivenom 2 h following the injection of venom [36].

## Figures and Tables

**Table 1:** Neutralizing antibody yield of plasma and purified serum.

	Protein Concentration (%)	Volume (mL)	Quantity (g)	Yield (%)
Plasma	14.06	150 mL	21.1	100
Purified serum	8.65	20 mL	1.73	8.2

**Table 2:** Effective dose (ED_50_) values for the antivenom developed against the venom of scorpions *Am*, *Bo*, and *Aah*.

	*Am*	*Bo*	*Aah*
ED_50_ (µL)	47.3 ± 0.33	51.86 ± 1.61	64.9 ± 1.95
ED_50_ (number of LD_50_ per mL of antivenom)	63.4	57.8	46.2

**Table 3:** Immunodetection of toxins fixed in brain and heart tissues in mice envenomed and neutralized by the developed antivenom.

	Group	Type of Staining	Type of Cell	Percentage
**Brain**	*Am* Venom	Cytoplasmic Membrane	- Nerve - Endothelial	70
	V *Am* + AV (2 h delay)	Cytoplasmic Membrane	- Nerve	40
	*Bo* Venom	Cytoplasmic Membrane	- Nerve	50
	V *Bo* + AV (2 h delay)	Cytoplasmic Membrane	- Nerve	30
	*Aah* Venom	Membrane	- Nerve - Endothelial	60
	V *Aah* + AV (2 h delay)	Membrane	- Nerve - Inflammatory	20
**Heart**	*Am* Venom	Cytoplasmic Membrane	- Myocardial - Endothelial	90
	V *Am* + AV (2 h delay)	Cytoplasmic Membrane	- Myocardial	50
	*Bo* Venom	Cytoplasmic Membrane	- Myocardial	60
	V *Bo* + AV (2 h delay)	Cytoplasmic Membrane	- Myocardial	30
	*Aah* Venom	Cytoplasmic Membrane	- Myocardial - Endothelial - Inflammatory	70
	V *Aah* + AV (2 h delay)	Cytoplasmic Membrane	- Myocardial	30

**Table 4:** Immunization schedule.

Bleeding Number	Days	Venom Dose (µg/Rabbit)	Nature and Volume of Adjuvant Used	Total Volume Injected (mL/Rabbit)	Number and Route of Injection Site
1	0	20	FCA (1.3 mL)	1	6 sites (SC)
2	7	20	FIA (1.3 mL)	1	3 sites (SC)
3	14	50	FIA (1.0 mL)	1	3 sites (SC)
4	21	100	FIA (1.3 mL)	1	3 sites (SC)
5	28	150	NaCl 0.9%	1	3 sites (SC)
6	35	200	NaCl 0.9%	1	3 sites (SC)
7	42	-	-	-	-

FCA: Freund’s Complete Adjuvant, FIA: Freund’s Incomplete Adjuvant, SC: Subcutaneous.

## Data Availability

The data that support the findings of this study are available from the corresponding author upon reasonable request.
